# Molecular conformational evolution mechanism during nucleation of crystals in solution

**DOI:** 10.1107/S2052252520004959

**Published:** 2020-04-24

**Authors:** Xin Li, Na Wang, Jinyue Yang, Yunhai Huang, Xiongtao Ji, Xin Huang, Ting Wang, Honghai Wang, Hongxun Hao

**Affiliations:** a National Engineering Research Center of Industrial Crystallization Technology, School of Chemical Engineering and Technology, Tianjin University, Tianjin 300072, People’s Republic of China; b School of Chemical Engineering, Hebei University of Technology, Tianjin 300130, People’s Republic of China; c Collaborative Innovation Center of Chemical Science and Engineering, Tianjin 300072, People’s Republic of China

**Keywords:** conformational polymorphism, nucleation, desolvation, molecular mechanisms, molecular conformations, intermolecular interactions

## Abstract

Nucleation of crystals from solution is fundamental to many natural and industrial processes. In this work, the molecular mechanism of conformational polymorphism nucleation and the links between the molecular conformation in solutions and in crystals were investigated in detail by using 5-nitro­furazone as the model compound. Different polymorphs were prepared, and the conformations in solutions obtained by dissolving different polymorphs were analysed and compared. The solutions of 5-nitro­furazone were proven to contain multiple conformers through quantum chemical computation, Raman spectra analysis, 2D nuclear Overhauser effect spectroscopy spectra analysis and molecular dynamics simulation. The conformational evolution and desolvation path was illustrated according to the ^1^H NMR spectra of solutions with different concentrations. Finally, based on all the above analysis, the molecular conformational evolution path during nucleation of 5-nitro­furazone was illustrated. The results presented in this work shed a new light on the molecular mechanism of conformational polymorphism nucleation in solution.

## Abstract 2

The results presented in this work give new insights into the molecular mechanism of crystallization nucleation for conformational polymorphic systems. It can be used to regulate conformational polymorphism.

## 1. Introduction   

Nucleation of crystals from solution is fundamental to many natural and industrial processes (Myerson & Trout, 2013 ▸). The molecular mechanism of nucleation has been extensively studied in recent years (Van Driessche *et al.*, 2018 ▸; Jehannin *et al.*, 2019 ▸; Chen *et al.*, 2019 ▸; Gebauer *et al.*, 2014 ▸; Erdemir *et al.*, 2009 ▸; Burton *et al.*, 2010 ▸; Davey *et al.*, 2002 ▸, 2013 ▸, 2015 ▸). However, the molecular assembly and evolution mechanism of the nucleation process is still not well understood. Many puzzles need to be solved to better understand the nucleation process, especially nucleation of conformational polymorphism (Nangia, 2008 ▸; Cruz-Cabeza & Bernstein, 2014 ▸; Cruz-Cabeza *et al.*, 2015 ▸; Yu *et al.*, 2000*a* ▸,*b* ▸).

Conformational polymorphism is the convergence of two central themes in structural chemistry: polymorphism and molecular conformation. There is great significance in the merging of the two into an important component of current molecular science (Cruz-Cabeza & Bernstein, 2014 ▸). Three aspects about the nucleation of conformational polymorphism have been studied systematically. (1) The influence of crystal structure on molecular conformation. Lattice energy minimization techniques were initially used for studying the influence of crystal forces on molecular conformation, and then were extended to investigate the influence of orientational and positional disorder on molecular conformation in trimorphic and isomorphic systems (Bernstein & Hagler, 1978 ▸; Hagler & Bernstein, 1978 ▸; Bar & Bernstein, 1982 ▸, 1984 ▸, 1985 ▸). (2) The effect of multiple molecular conformations in solution on the crystallization process. The crystallization kinetics from solutions containing multiple conformers have been systematically studied. A model of crystal growth was used and improved to evaluate the effect of conformation on nucleation difficulty and was proved with experimental results (Derdour *et al.*, 2011 ▸, 2012 ▸; Derdour & Skliar, 2012 ▸, 2014 ▸). (3) Are there some links between the molecular conformation in solutions and in crystals? (Zeglinski *et al.*, 2018 ▸) Extensive studies in recent years showed that the molecular conformation in solutions could also appear in crystals, while others obtained doubtful results.

In terms of the third aspect, the relationship of solvent, solute conformation and solution structure was studied through crystal nucleation of tolbutamide in solutions (Zeglinski *et al.*, 2018 ▸). The investigating results showed that tolbutamide conformations in solutions of iso­propanol, ethyl acetate and aceto­nitrile also existed in crystals. However, one tolbutamide conformation with intramolecular hydrogen bonds in the solution of toluene did not appear in crystals. And the influence of solvents on the nucleation process was reflected in the desolvation process and the molecular conformation. Another study of evaporative crystallizations of ethenzamide in 18 solvents (Back *et al.*, 2012 ▸) showed that the crystal structure of ethenzamide is built from a high-energy conformer rather than a low-energy conformer. It also indicated that the mechanism of self-assembled crystallization of different molecular conformations was not clear. In addition, an investigation about additive mediated *syn*-*anti* conformational tuning at nucleation to capture elusive polymorphs (Gawade *et al.*, 2016 ▸) showed that the solution was a multi-conformational solution regardless of the presence or absence of additives. Additives did not directly change the conformations of solute molecules in the solution but provided a template through the interaction between solutes and additives, which is more conducive to the aggregation of conformations in metastable form and the formation of metastable crystals. Therefore, molecular conformation evolution in the process of nucleation and crystallization is not a simple process but a complex process depending on the properties of solvent and solute.

Moreover, different results or conclusions were reported even for the same substance. Tolfenamic acid is a typical case. When crystallizations were conducted in ethanol under different supersaturation conditions (Mattei & Li, 2011 ▸, 2012 ▸), the solute molecular conformations rearranged to form hydrogen-bonded dimers in the solution as the concentration increased. Therefore, the metastable form appeared at low supersaturation while the stable conformational polymorph containing dimers appeared at high supersaturation. This indicated that there were direct connections between the conformation in solutions and in crystals. Their further research (Mattei & Li, 2014 ▸) illustrated the underlying cooperation of molecular conformation, intermolecular interaction and the nucleation of polymorph through molecular dynamics and quantum mechanics methods. However, when crystallizations were conducted in more solvents and wider supersaturation ranges (Du *et al.*, 2015 ▸), the results were contrary to previous conclusions. It was found that there were no favoured conformations in solution irrespective of the existence of dimers. Another study (Tang *et al.*, 2017 ▸) showed that both ‘twist-like’ and ‘planar-like’ conformation existed in solution, and there were no links between conformations in solutions and in crystals. Therefore, if we want to find the correlation between solution chemistry and crystallization to control conformational polymorphism, it is necessary to find out what conformers exist in solutions and understand how conformations change during the crystallization process.

**Figure 1:**
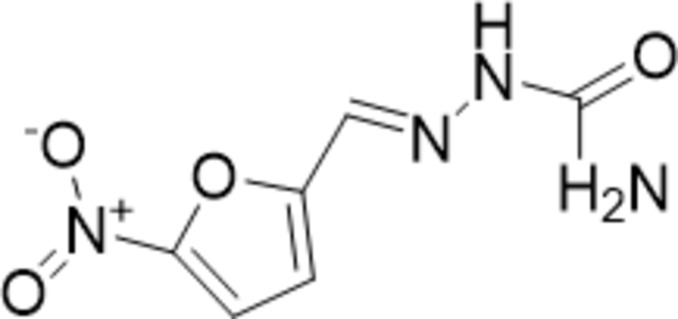
Chemical structure of 5-nitro­furazone.

From reported studies, it can be found that there was no consensus about the link between conformations in solutions and in crystals. Hence, different opinions exist. It is important to understand the conformational evolution path during nucleation since the conformational polymorphism can be regulated through inhibiting or promoting the formation of corresponding conformation if we know the conformational evolution path. Thus, in this work, the molecular conformational evolution mechanism during nucleation of crystals in solution was investigated by using 5-nitro­furazone [Fig. 1 ▸, CAS number 59-87-0) as the model compound. ^1^H NMR spectra (Back *et al.*, 2012 ▸), nuclear Overhauser effect spectroscopy (NOESY) (Tang *et al.*, 2017 ▸), Raman spectra (Simone & Nagy, 2015 ▸), quantum chemical computation (Du *et al.*, 2015 ▸) and molecular dynamics (MD) simulation have proven to be reliable methods for investigating molecular conformation in solutions and crystals. Therefore, these methods will be used in this work.

Three polymorphs of 5-nitro­furazone have been reported: α form (conformational polymorphism, CCDC reference 1292340, Fig. S1), β form (CCDC reference 1444950, Fig. S2) and γ form (CCDC reference 1444951, Fig. S3) (Olszak *et al.*, 1994 ▸; Pogoda *et al.*, 2016 ▸). After the preparations of the three polymorphs, cooling crystallization experiments in *N*,*N*-di­methyl­formamide (DMF), di­methyl sulfoxide (DMSO) and *N*,*N*-di­methyl­acetamide (DMAC) with different polymorphs as raw materials were conducted first. Then, the molecular conformations in different solutions were analysed through Raman spectra, NOESY, quantum chemical computation and MD simulation. Next, on the basis of the above analysis, ^1^HNMR spectra of different concentrations were employed to deduce the key step in the conformational evolution process. Finally, the molecular conformational evolution path during nucleation of 5-nitro­furazone was proposed and illustrated based on the obtained data.

## 2. Experimental section and simulation methods   

### 2.1. Materials   

5-nitro­furazone (CAS No. 59-87-0, >99% purity) form β was purchased from TCI-E7CCN and used without further purification. The analytical reagent grade (the mass purities > 99.5%) DMF, DMSO and DMAC were purchased from Jizhun Chemical Technology Co. Ltd.

### 2.2. Preparation of polymorphs   

5-nitro­furazone form β was used as the starting material to produce the other two forms through suspension crystallization. 5-nitro­furazone form α was produced in DMF solvent (12.00 g of 5-nitro­furazone form β and 100.00 g of DMF) at 30°C. 5-nitro­furazone form γ was produced in DMF solvent (12.00 g of 5-nitro­furazone form β and 100.00 g of DMF) at 50°C.

The suspension crystallization products were confirmed by three methods. (1) The powder X-ray diffraction (PXRD) data were obtained on a Rigaku D/max-2500 (Rigaku, Japan) using Cu Kα radiation (1.5405 Å), with a step size of 0.02° and a scanning rate of 0.067° s^−1^ over a diffraction angle (2θ) range of 5–50°. (2) The Fourier transform infrared spectra (FTIR) of the products were collected using a Bruker Alpha FTIR-ATR (attenuated total reflection) instrument with 4 cm^−1^ resolution and 32 scans per spectrum at 4000–400 cm^−1^ range. (3) The Raman spectra were obtained on the RamanRXN2 HYBRID analyzer (Raman, Kaiser Optical Systems Inc., USA) which is equipped with both a *PhAT* probe head (for non-contact measurements, for the detection of crystal production here) and an MR probe head (for direct measurements, for the detection of solution) at 1800–200 cm^−1^ range. And the purity of form γ and form α was detected by a UV-2600 spectrophotometer (SHIMADZU, Japan). The method and standard curve have been described in our previous work (Li *et al.*, 2019 ▸).

### 2.3. Solubility determination   

The UV spectroscopic method was used to determine the solubility of 5-nitro­furazone in DMF, DMSO and DMAC. At first, an excess amount of 5-nitro­furazone was put into a water-jacketed glass vessel (80 ml) with almost 60 ml of solvents and a thermostat (Xianou Laboratory Instrument Works Co. Ltd, Nanjing) with an accuracy of ±0.1 K was used to keep the system at 20°C. The suspended solution was agitated adequately by a magnetic stirrer to guarantee the solid–liquid equilibrium. Next, the solution was kept at the same temperature without agitation to ensure settle down of the undissolved solids. And then, the upper clear saturated solutions were withdrawn by syringes with organic membrane filters (0.22 µm, Tianjin Legg Technology Co. Ltd, Tianjin, China) and were then diluted to the concentration range that was appropriate for UV assay. Finally, the concentration was determined by the UV-2600 spectrophotometer (SHIMADZU, Japan). The method and standard curve have been described in our previous work (Li *et al.*, 2019 ▸).

### 2.4. Crystallization experiments   

The crash-cooling crystallization experiments of 5-nitro­furazone were conducted in DMF, DMSO and DMAC. These experiments were carried out using an 80 ml jacketed vessel with a magnetic stirring at 200 r min^−1^. Different 5-nitro­furazone forms were added into 20 g of solvent under a supersaturation of 1.3 to prepare the solutions. The solutions were kept at 70–90°C for 1 h to ensure that the crystals were completely dissolved. The solutions were withdrawn by syringes with organic membrane filters (0.22 µm, Tianjin Legg Technology Co. Ltd, Tianjin, China) and then transferred to the jacketed vessel pre-set at 20°C (Xianou Laboratory Instrument Works Co. Ltd, Nanjing). After stirring for 0.5 h to increase the yield, the crystals were filtered and characterized by PXRD, Raman and FTIR.

### 2.5. Quantum chemical computation   

**Figure 2:**
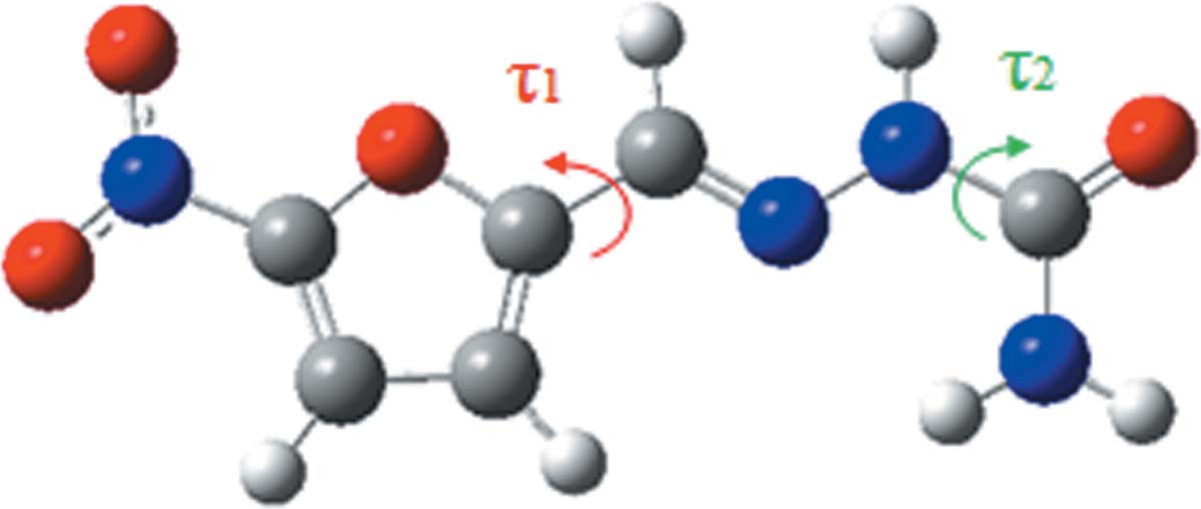
Rotatable single bonds of 5-nitro­furazone related to conformational change.

For the potential energy surface (PES) scan, the conformer in 5-nitro­furazone form β was extracted and its geometry was optimized (named conformer B). Then, the PES of 5-nitro­furazone conformer B with dihedral angles τ_1_ and τ_2_ (Fig. 2 ▸) was generated by scanning for 18 steps with a step length of both 10° and −10° for τ_1_ and τ_2_. All calculations were performed in the gas-phase and DMSO environment at the M06-2X/6-31 + G(d,p) level of theory with *GAUSSIAN09* (Frisch *et al.*, 2009 ▸).

For the computation of molecular geometries and energies, the various conformers at the local energy-minimum points of the PES were extracted and then calculated in *GAUSSIAN09* (Frisch *et al.*, 2009 ▸). The various conformers were geometrically optimized in gas-phase and different solvent environments with SMD (universal solvent model based on the solute electron density) (Tomasi *et al.*, 2005 ▸; Marenich *et al.*, 2009 ▸) implicit solvation models (with the force constants being calculated at all points and tight convergence criteria). Then, the conformer energies in gas-phase and different solvent environments were calculated with SMD solvation models. All calculations were performed at the M06-2X-D3/6-311 + G(d,p) level of theory.

### 2.6. Raman spectra of solution and its dilution process   

Raman spectroscopy has proven to be an effective method for investigating the polymorphic outcome in cooling crystallization (Simone & Nagy, 2015 ▸). To detect the Raman spectra, 20 ml of DMSO and excessive amounts of different 5-nitro­furazone forms were added into a glass bottle at 20°C to prepare the suspensions. The suspensions were stirred at 200 r min^−1^ with a magnetic stirring for 1 h at 20°C to ensure sufficient dissolution. The suspensions were withdrawn by syringes with organic membrane filters (0.22 µm, Tianjin Legg Technology Co. Ltd, Tianjin, China) to obtain the clarified saturated solutions. Each of the saturated solution was put into a 10 ml glass bottle for Raman analysis. Then, the saturated solution was diluted six times and each diluted concentration was 0.5 times lower than the previous diluted concentration. The Raman spectra of all the diluted solutions were collected.

### 2.7. Nuclear Overhauser effect spectroscopy   

2D NOESY experiments were carried out for 5-nitro­furazone solutions in DMSO-d6 at room temperature by using a 600 MHz Bruker AVANCE III NMR spectrometer. 2D NOE spectra were measured with a standard pulse in DMSO-d6 for both F1 and F2 dimensions. The number of F1 increments was 256, each with 65 536 data points in the F2 dimension. The NOE mixing time was optimized to 0.8 s by measuring NOE buildups. The number of scans and dummy scans were set to be 16 and 2, respectively.

### 2.8. Molecular dynamics simulation   

All simulations were performed with Materials Studio 7.0 on a Dell T7910 workstation. The Amorphous Cell module was used to construct the cubic cells. Every cubic cell contained 1000 solvent molecules and a certain number of solute molecules in conformer E (the conformer with the highest energy) according to solubility of different polymorphs in DMSO. The Forcite module was used for the MD simulation, in which the COMPASS II (condensed-phase optimized molecular potentials for atomistic simulation studies, a type of class II *ab initio* consistent force field) force field (Sun *et al.*, 2016 ▸) was used to describe the interactions throughout the simulations at a fully atomistic level. The Smart approach, which combines the conjugate-gradient and steepest-descent methods, was applied in the energy-minimization procedure to accelerate the computation.

First, 100 000 steps of molecular mechanics (MM)-based geometry optimization were applied to the box to eliminate irrelevant contacts. Then, the NPT ensemble MD simulation was performed at the experimental temperature to ensure a well relaxed system and to achieve the equilibrium. During the data sampling, the Nosé thermostat and Berendsen barostat were used to control the temperature and pressure, respectively. The simulation time was set at 50 ps and the time step was set to 1 fs for each MD process. Then, radial distribution functions (RDFs) were used to analyse the conformations in the solutions.

### 2.9. ^1^HNMR spectra of different concentrations   

^1^HNMR spectra of different concentrations were detected using a 600 MHz Bruker AVANCE III NMR spectrometer. First, 5 ml of DMSO-d6 and excessive 5-nitro­furazone (form α were chosen as an example) were added into a glass bottle at 20°C to prepare the suspension. The suspension was then stirred at 200 r min^−1^ with a magnetic stirring for 30 min at 20°C to ensure sufficient dissolution. The suspension was withdrawn by syringes with organic membrane filters (0.22 µm, Tianjin Legg Technology Co. Ltd, Tianjin, China) to obtain the clarified saturated solution. Different contents (*e.g.* 500, 440, 380, 320, 260, 200, 140, 100, 80, 30 and 20 µl) of the saturated solution were put into different NMR tubes. And then, a certain amount of DMSO-d6 was added into the tube of 500 µl for^1^HNMR analysis.

## 3. Results and discussion   

### 3.1. Characterization and identification of the crystal products   

**Figure 3:**
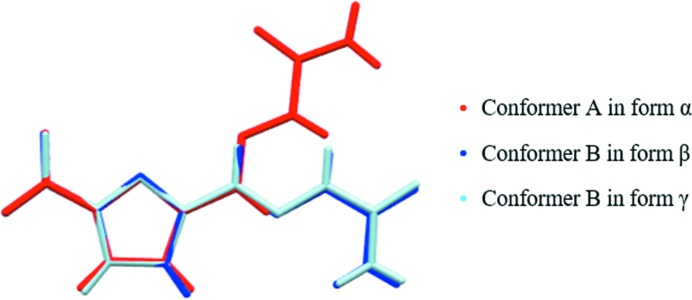
Conformations in three crystalline forms of 5-nitro­furazone.

In order to study the solution structure and the crystal structure, the single-crystal data of the three forms were found in the CCDC and are shown in Figs. S1–S3. Obvious differences in intermolecular interactions (shown in Table 1 ▸) and molecular conformation (shown in Fig. 3 ▸) can be observed. All these three forms form an extended chain structure firstly, and then the chain structure expands into a planar structure by NO_2_⋯H—C connection between the two chains. Finally, a 3D crystal structure is formed by hydrogen bond and π⋯π stacking interaction. The PXRD patterns of different forms were calculated through these single-crystal data with Mercury 3.10.2 and were used to identify the forms of crystal products in the following investigations.

**Figure 4:**
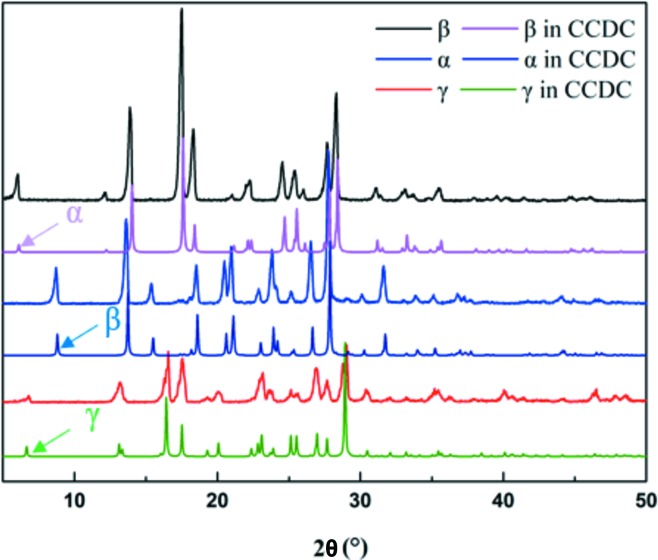
PXRD patterns of 5-nitro­furazone prepared in a laboratory and found in the CCDC.

**Figure 5:**
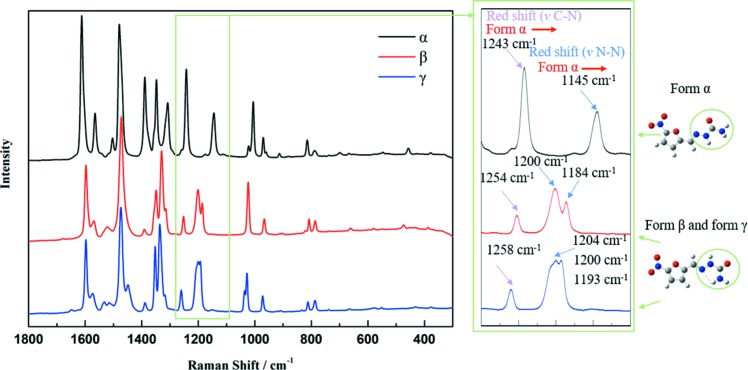
Raman spectra of each form prepared in a laboratory.

**Figure 6:**
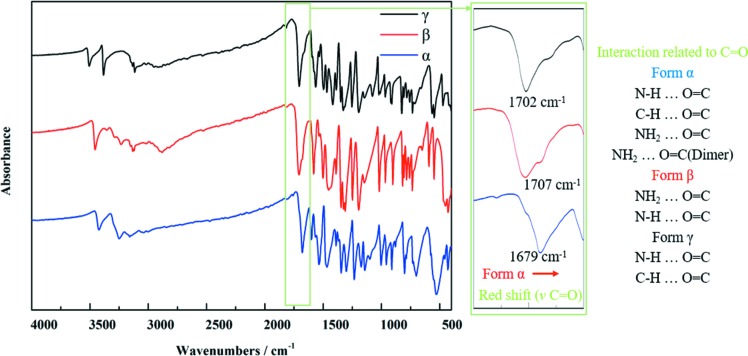
FTIR spectra of each form prepared in a laboratory.

The identification of the raw material (form β) and prepared form α and form γ were confirmed by their PXRD patterns, FTIR spectra and Raman spectra. As shown in Fig. 4 ▸, the experimental PXRD patterns are consistent with the computational PXRD patterns from the single-crystal data from the CCDC, thus confirming their crystal forms. The Raman spectra and the FTIR spectra are shown in Fig. 5 ▸ and Fig. 6 ▸, respectively. The Raman and FTIR spectra of form β and form γ are consistent with the literature data (Pogoda *et al.*, 2016 ▸). Although the Raman and FTIR spectra of form α are not found in the literature, they show obvious differences with the other two forms. In Raman spectra, because of the intra NH_2_⋯N hydrogen bonds in form β and form γ, the stretching of N—N (*v* N—N, where *v* represents the stretching frequency) of form α (1145 cm^−1^) and the stretching of C—N (*v* C—N) of form α (1243 cm^−1^) show a red shift compared with form β (*v* N—N = 1184 and 1200 cm^−1^; *v* C—N = 1254 cm^−1^) and form γ (*v* N—N = 1193, 1200 and 1204 cm^−1^; *v* C—N = 1258 cm^−1^). These differences can be used to identify different crystal forms and their approximate molecular conformations. From the FTIR spectra shown in Fig. 6 ▸, since the interaction related to C=O in form α is obviously stronger than that in form β and form γ, the stretching of C=O (*v* C=O) of form α (1679 cm^−1^) shows a red shift compared with form β (1702 cm^−1^) and form γ (1707 cm^−1^). It can also be used to identify crystal forms and estimate their molecular interactions.

**Figure 7:**
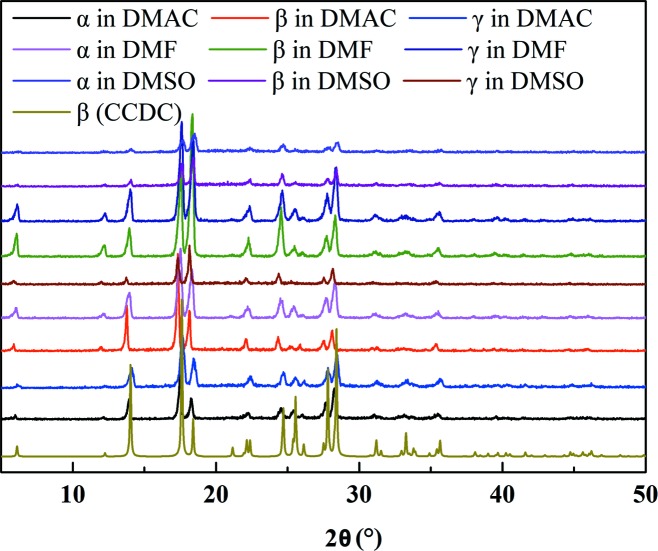
PXRD patterns of crash-cooling experimental products and form β in the CCDC.

The PXRD patterns of products obtained from crash-cooling experiments by using different forms as raw materials (mass purity of each form: >99.0%) are shown in Fig. 7 ▸. It can be seen that all the products of 5-nitro­furazone obtained from DMF, DMSO and DMAC under the supersaturation ratio of 1.3 are form β. This indicates that the polymorph of crystallization products was not affected by the form of raw materials during crash-cooling crystallization and no direct connections between the conformation in raw crystals and the conformation in product crystals were found. To understand what factors will affect the conformation of crystallized products, it is essential to analyse the solution structure. In addition, it is well known that different crystal forms of the same pharmaceutical compound might have different bioavailability (Simone & Nagy, 2015 ▸), such as chloramphenicol palmitate (Mishra *et al.*, 2013 ▸), ritonavir, rotigotine and ranitidine hydro­chloride (Neumann & Jacco, 2018 ▸). If the conformations of dissolved different forms are completely the same, it will be difficult to explain the different bioavailability when the pharmaceutical compound is dissolved in water for injection or dissolved in blood directly for human use. To verify this, the solution thermodynamics and solution structures of different forms were subsequently investigated.

### 3.2. Solubility of different forms   

The raw material (5-nitro­furazone form β) and the newly prepared products (form α and form γ) were used to collect solubility data at 20°C. As shown in Table 2 ▸, the solubility order is: form β > form α > form γ. This indicates that the thermodynamic stability order is: form β < form α < form γ, which is consistent with the suspension crystallization experiments. Combining the crash-cooling crystallization experiments and suspension crystallization experiments of 5-nitro­furazone, it can be found that the thermodynamic metastable form β would crystallize out first and then it can transform into the relatively stable form α (30°C) and form γ (50°C) under different temperatures, which follows the Ostwald’s rule (Threlfall, 2003 ▸). Moreover, the different solubility data of these three forms also indicate the existence of some subtle differences in solutions obtained by dissolving different polymorphs.

Since crystallization phenomena are similar in the three solvents, DMSO was selected as an example to analyse the initial solution structures. According to the solubility data, the molecular numbers of form α, form β and form γ in 1000 DMSO molecules are 78, 94 and 76, respectively. And these molecular numbers are used in the next MD simulations to analyse the solution structures.

### 3.3. Conformation analysis   

**Figure 8:**
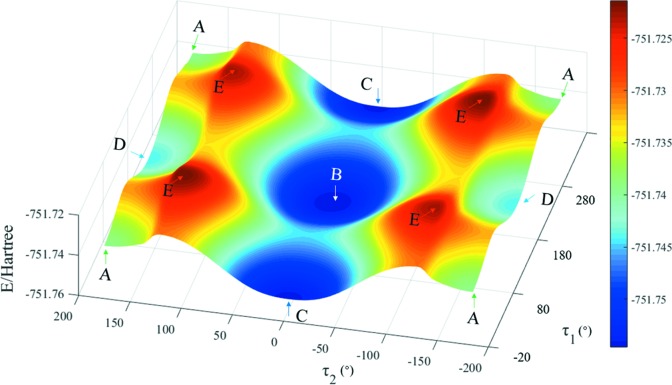
The PES of optimized conformer B of 5-nitro­furazone in gas phase (18 steps were scanned with a step length of both 10° and −10° for τ_1_ and τ_2_).

The PES scan of 5-nitro­furazone about τ_1_ and τ_2_ is shown in Fig. 8 ▸. There are nine local energy-minimum points in the PES, which are related to four planar conformers [shown in Figs. 8 ▸(*a*), 8(*b*), 8(*c*) and 8(*d*)]. Conformer A is the same as the conformer in 5-nitro­furazone form α whilst conformer B is the same as the conformer in 5-nitro­furazone form β and form γ. Conformer C and conformer D are the conformers at another two local energy-minimum points, which are not found in the crystalline structures. There are four energy-maximum points in the PES, which are related to the same twist conformer, named conformer E [shown in Fig. 8 ▸(*e*)]. These data indicate that the energies of planar structures are lower while the energies of twist structures are relatively higher. Therefore, during the conformational transformation process, when the two angles twist close to a planar structure, the conformational energy will become lower. Likewise, when the two dihedral angles are far away from a planar structure, the conformational energy will become relatively higher.

**Figure 9:**
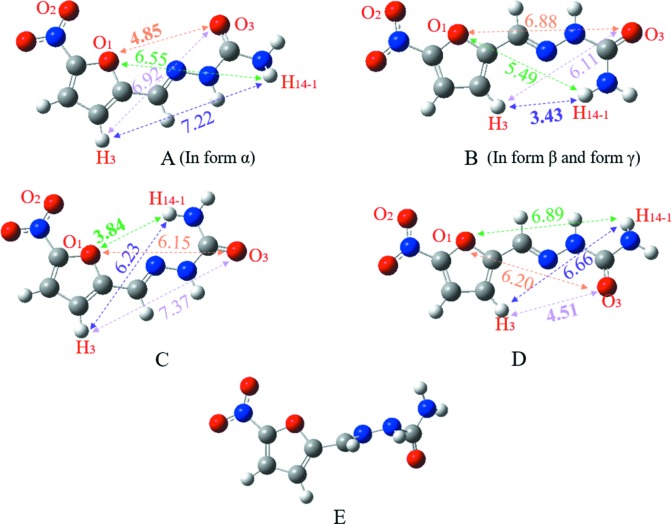
The conformers at the local energy-minimum points (A, B, C and D) with their characteristic distances and the conformer at the energy-maximum points (E) of the PES.

The various conformers at the local energy-minimum points were geometry optimized in gas-phase and different solvent environments with SMD solvation models. And then, distances between different atoms in different conformations were measured (as shown in Fig. 9 ▸ and Table 3 ▸). It can be seen that the distance of H_3_–H_14–1_ in conformer B is 3.43 Å, while the distance of H_3_–H_14–1_ is >6.23 Å in conformers A, C and D. Therefore, the distance of H_3_–H_14–1_ can be used as the characteristic distance of conformer B. Similarly, the distances of O_3_–O_1_ (A = 4.85 Å, the others are >6.15 Å), H_14–1_–O_1_ (C = 3.84 Å, the others are >5.49 Å) and H_3_–O_3_ (D = 4.51 Å, the others are >6.11 Å) can be used as the characteristic distances of conformers A, C and D, respectively. The characteristic distance is also used in the next NOESY analysis and MD simulation analysis to determine whether the conformers exist in the solution or not.

The conformer energies in gas-phase and different solvent environments with SMD solvation models were calculated after geometric optimization. As shown in Table 4 ▸, the conformational energy of conformer B is the lowest in gas phase, while the conformational energy of conformer C is the lowest in solvent environments. The conformational stability order in gas phase is conformer B (in form β and form γ) ≃ conformer C > conformer D > conformer A (in form α), while the conformational stability order in SMD solvation models is conformer C ≃ conformer B > conformer A ≃ conformer D.

**Figure 10:**
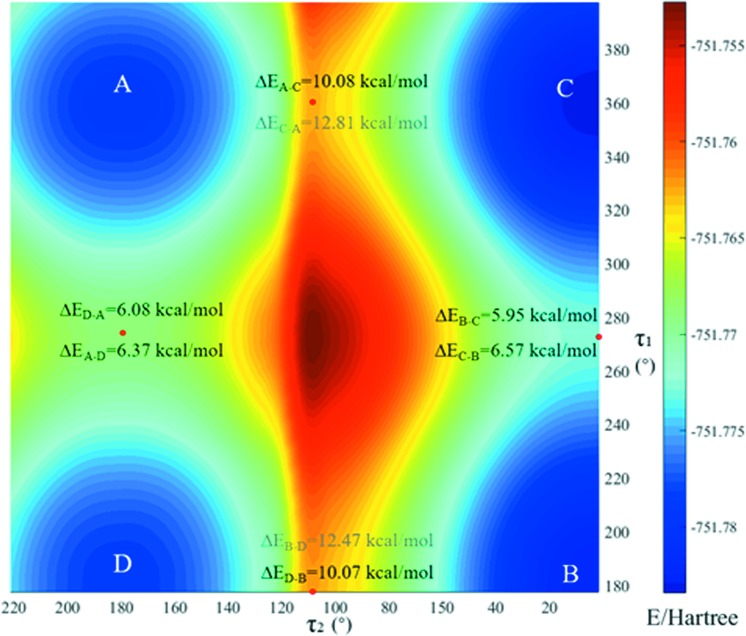
The PES of optimized conformer B of 5-nitro­furazone in DMSO (22 steps were scanned with a step length of 10° for τ_1_ and τ_2_).

Moreover, to investigate the difficulty of conformational transformation in solution, the PES of 5-nitro­furazone in DMSO solvent was scanned for 22 steps with a step length of 10° for two dihedral angles, by using optimized conformer B as the starting conformation. As shown in Fig. 10 ▸, the conformational energy barriers of the inter-transformation between conformers B and C and the inter-transformation between conformers A and D are lower than 10 kcal mol^−1^ while the conformational energy barriers of the inter-transformation between conformers A and C and the inter-transformation between conformers D and B are higher than 10 kcal mol^−1^. According to the study of Derdour & Skliar (2014 ▸), if the energy barrier of conformational change in solution is higher than 10 kcal mol^−1^, conformers in solutions will have a relatively long half-life. Therefore, the inter-transformation between conformers B and C and the inter-transformation between conformers A and D are easy to occur, and the inter-transformation between conformers A and C and the inter-transformation between conformers D and B are difficult to occur in the solution.

### 3.4. Conformations in the solution and the crystallization process   

**Figure 11:**
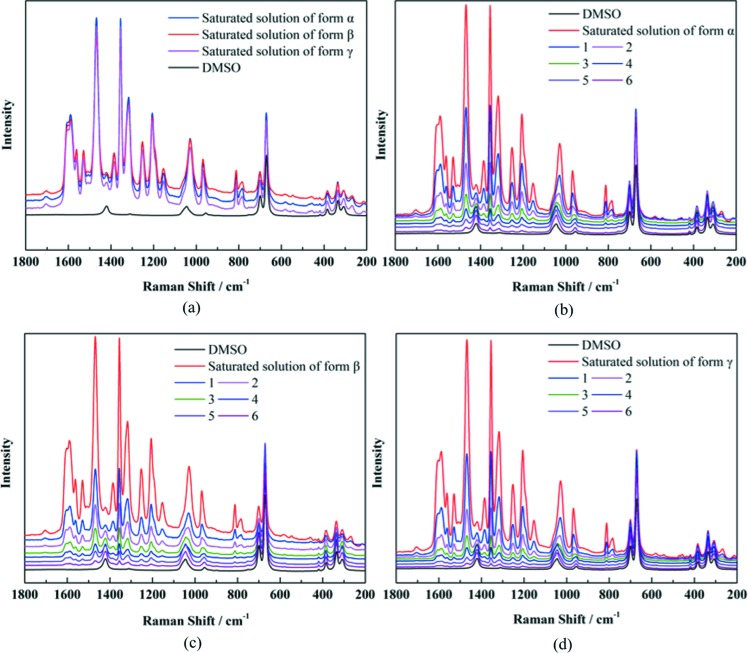
(*a*) Raman spectra of saturated solution of different forms in DMSO. (*b*) Raman spectra of saturated solution and its dilution process of form α in DMSO. (*c*) Raman spectra of saturated solution and its dilution process of form β in DMSO. (*d*) Raman spectra of saturated solution and its dilution process of form γ in DMSO. (The saturated solution was diluted six times and the diluted concentration was 0.5 times lower than the previous diluted concentration.)

Raman spectra of saturated solutions (to each form) and their dilution processes are shown in Fig. 11 ▸. It can be seen that no obvious Raman spectra differences exist among solutions obtained by dissolving different forms (also with different concentrations). This indicates that all the solutions obtained by dissolving different forms might be similar.

**Figure 12:**
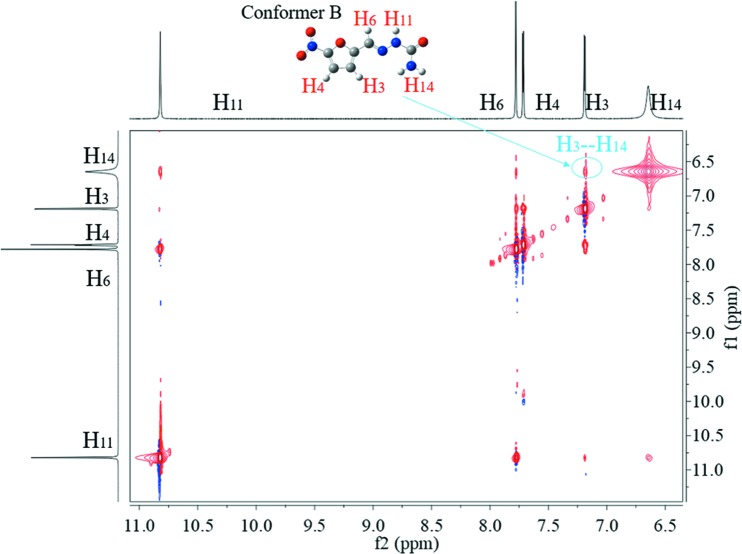
The 2D NOESY of form α saturated solution in DMSO at 20°C.

To further dig into the existing forms of 5-nitro­furazone, 2D NOESY was used to detect the NOE between different protons to deduce which conformers are contained in the solutions. As shown in Figs. 12 ▸, 13 ▸ and 14 ▸, the NOE cross peak of H_3_ and H_14_ appears in three forms. Combining the above data of the characteristic distance of different conformers, the distance of H_3_ and H_14_ in conformer B is 3.43 Å, while others are >6.23 Å. And the NOE appears when the space distance is closer than 5 Å. Therefore, the conformer B exists in DMSO solutions of all three crystalline forms at 20°C.

**Figure 15:**
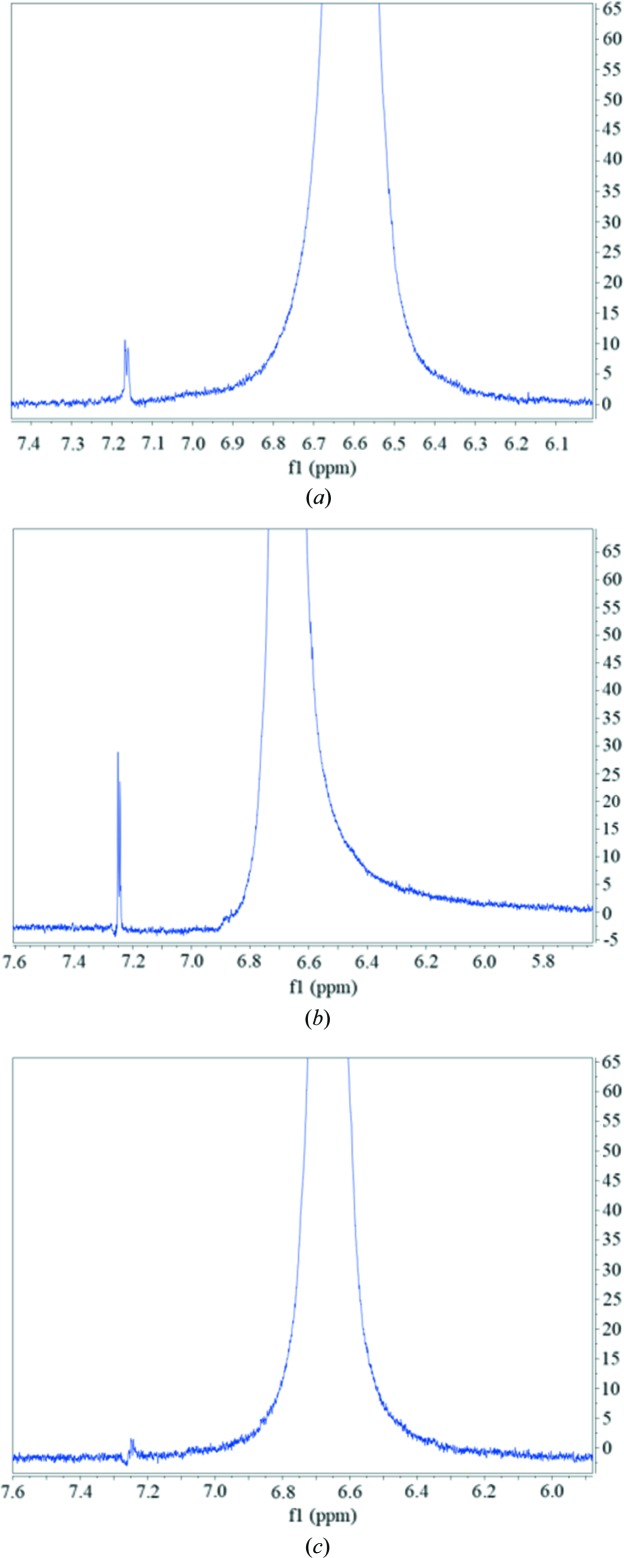
The 1D NOE of saturated solution in DMSO at 20°C. (*a*) Form α, (*b*) form β and (*c*) form γ.

It can also be seen that the NOE of H_3_ and H_14_ in saturated solution of form γ is not as obvious as those in saturated solutions of form α and form β, although form γ crystal contains conformer B and is the most stable form. To further verify the intensity of NOE between H_3_ and H_14_, the 1D NOE spectra were detected. As shown in Fig. 15 ▸, the NOE strengths of H_3_ and H_14_ of different solutions are obviously different, which are consistent with 2D NOESY results. Because H_3_ and H_14_ are within the same molecule, the different strengths of NOE were not caused by different solute concentrations. The difference in NOE strengths indicates that the conformations are not completely the same in these solutions. Some kind of differences should exist. Since it is impossible so far to identify, MD simulation was conducted.

**Figure 16:**
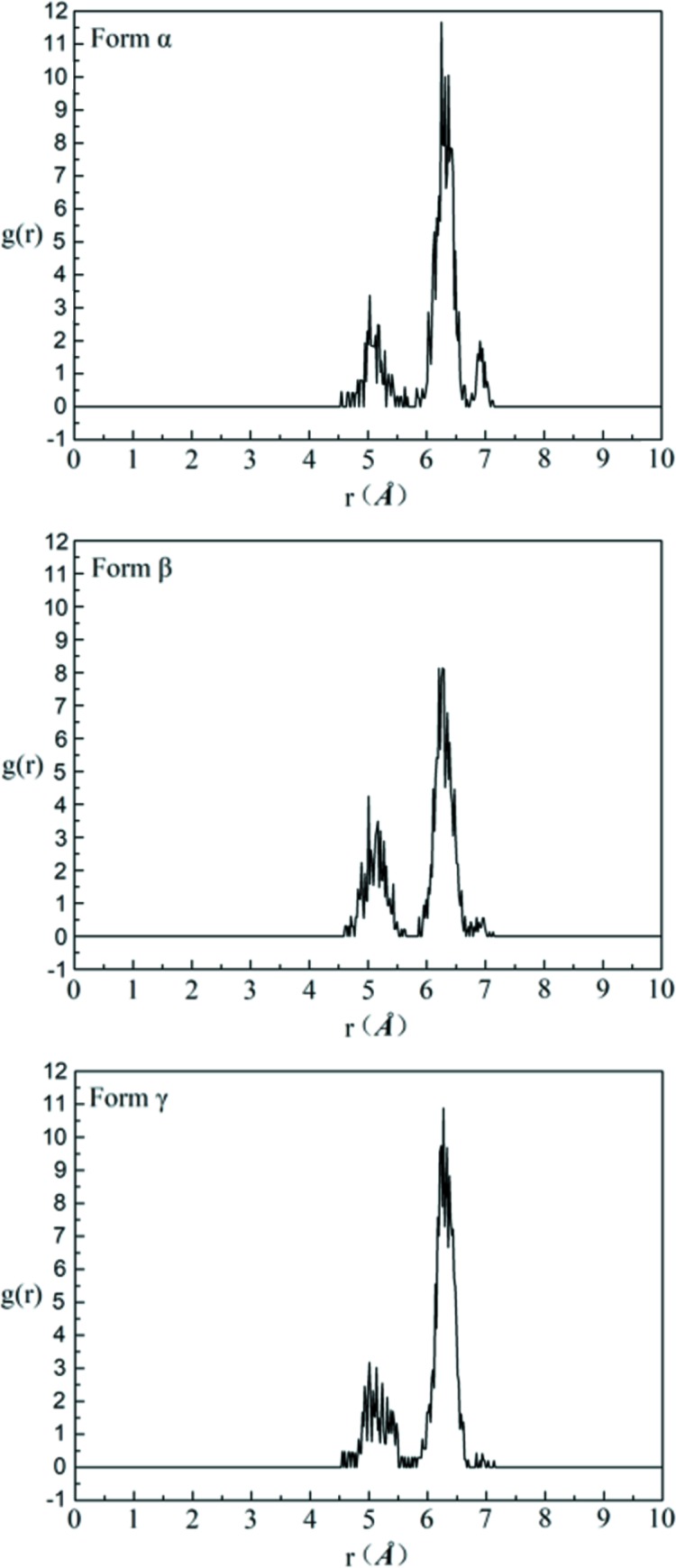
The RDFs of O_3_–O_1_ of saturated solutions of form α, form β and form γ in DMSO at 20°C.

**Figure 17:**
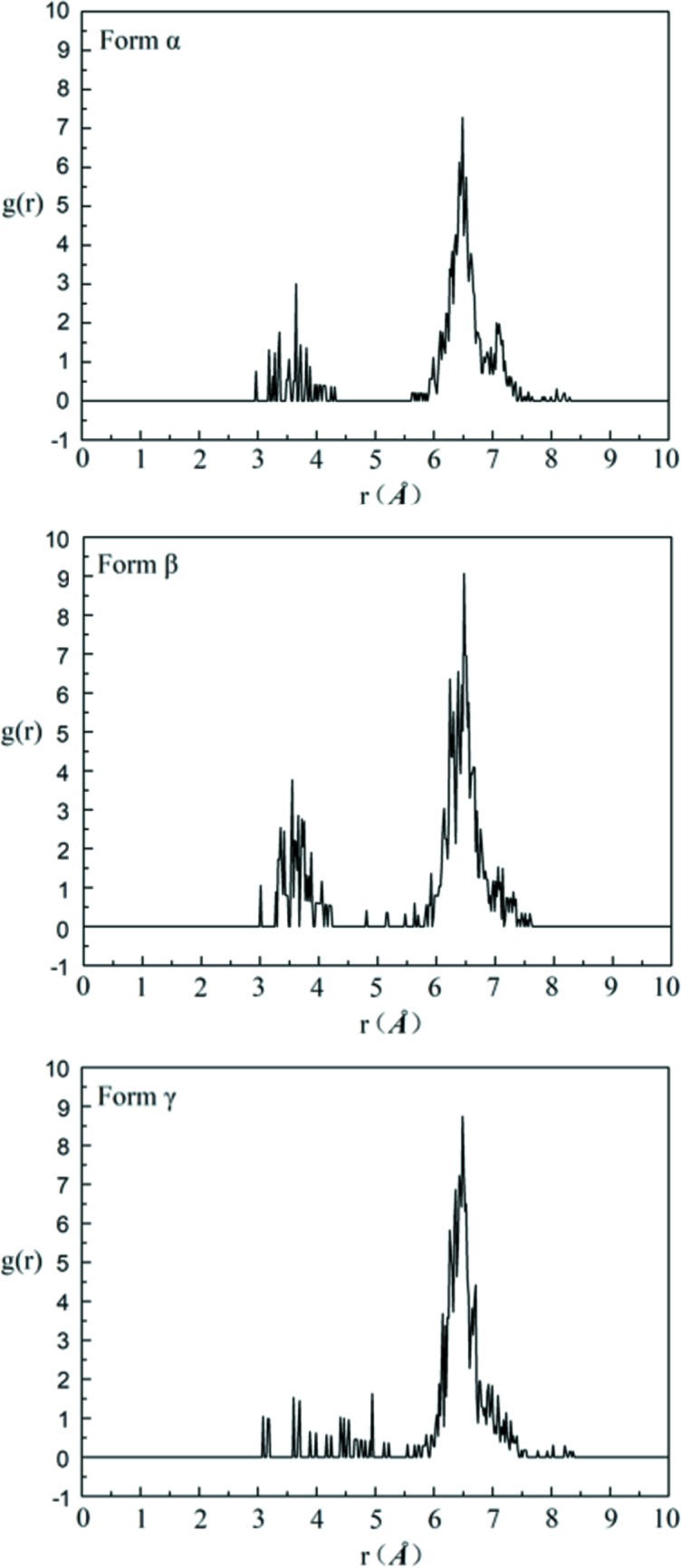
The RDFs of H_3_–H_14–1_ of saturated solutions of form α, form β and form γ in DMSO at 20°C.

**Figure 18:**
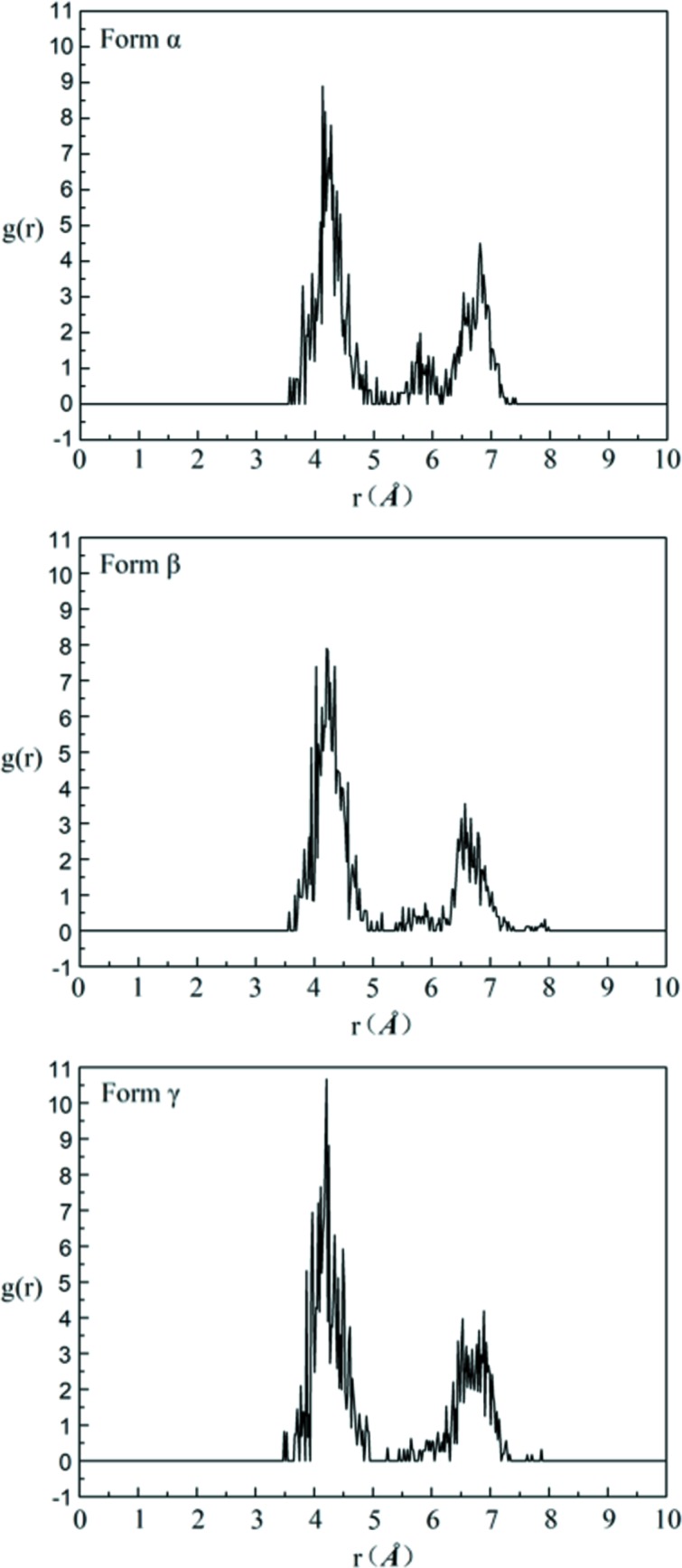
The RDFs of H_14–1_–O_1_ of saturated solutions of form α, form β and form γ in DMSO at 20°C.

**Figure 19:**
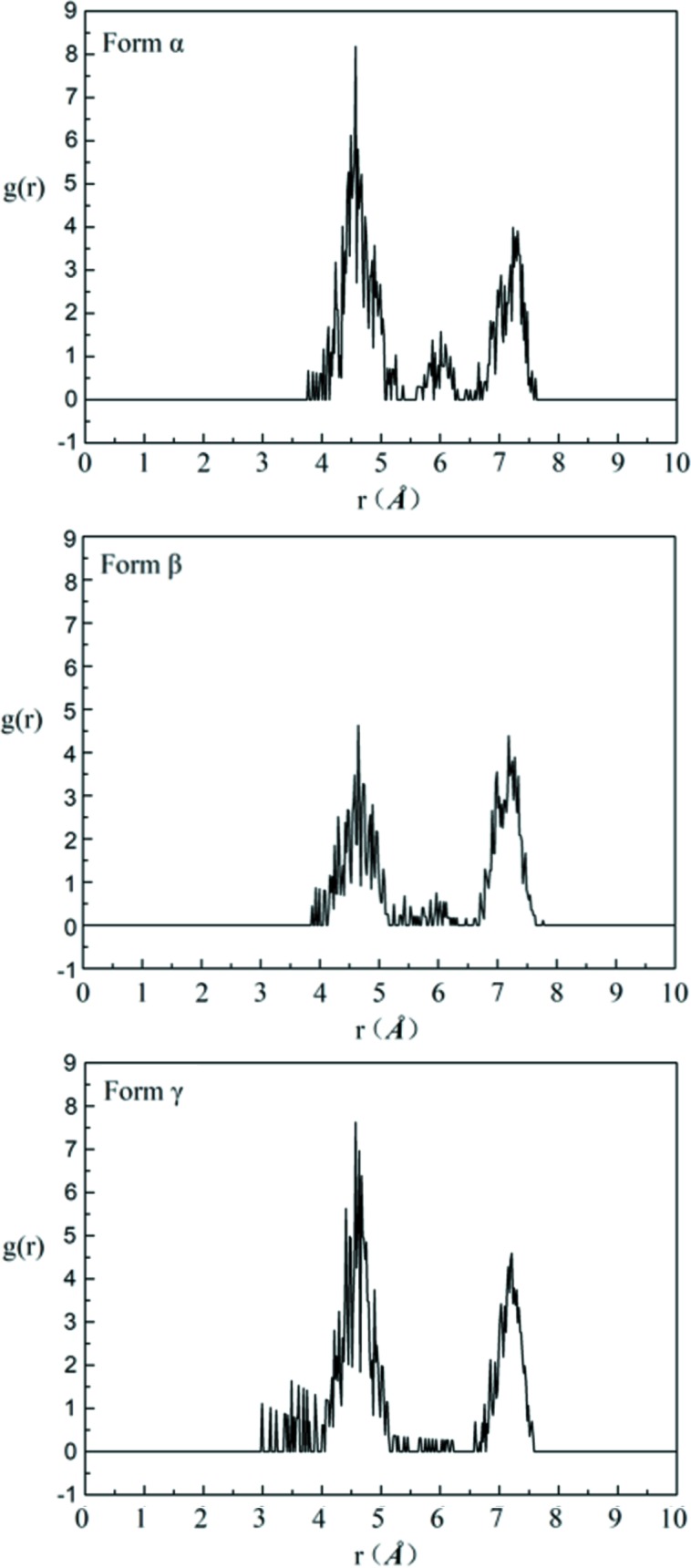
The RDFs of H_3_–O_3_ of saturated solutions of form α, form β and form γ in DMSO at 20°C.

Therefore, 1000 DMSO molecules and 78, 94 and 76 solute molecules were employed to construct an amorphous cell and to perform MD simulation for form α, form β and form γ, respectively. The four RDFs which correspond to the characteristic distances of four conformers in each solution are analysed after MD simulation by combining with the characteristic distances of different conformers. And the results of the RDFs are shown in Figs. 16–19.

Firstly, the RDFs of O_3_–O_1_, which reflect the existence of conformer A (characteristic distance of 4.85 Å, the others are >6.15 Å) in solutions, are shown in Fig. 16 ▸. There is a distribution at 4.85 Å and a local maximum point at ∼5.0 Å in each solution, indicating the existence of conformer A in each solution.

The RDFs of H_3_–H_14–1_, which reflect the existence of conformer B (characteristic distance of 3.43 Å, the others are >6.23 Å) in solutions, are shown in Fig. 17 ▸. There is a distribution at 3.43 Å and a local maximum point at ∼3.5 Å, which indicates the existence of conformer B. The peak at ∼3.5 Å for solutions obtained by dissolving form α and form β is lower and more dispersive than that for solutions obtained by dissolving form γ, which indicates that conformer B in the saturated solution of form γ is less than that in the saturated solutions of the other two forms. This shows a good consistence with the 2D NOESY results.

The RDFs of H_14–1_–O_1_, which reflect the existence of conformer C (characteristic distance of 3.84 Å, the others are >5.49 Å), are shown in Fig. 18 ▸. There is a distribution at 3.84 Å and a local maximum at ∼4.1 Å, which indicates the existence of conformer C. The distribution at ∼3.84 Å in saturated solution form γ is obviously higher than that in the saturated solutions of the other two forms. This indicates that the solution obtained by dissolving form γ contains more conformation C than the solutions obtained by dissolving the other two forms.

Finally, the RDFs of H_3_–O_3_, which reflect the existence of conformer D (characteristic distance of 4.51 Å, the others are >6.11 Å), are shown in Fig. 19 ▸. There is a maximum distribution at ∼4.51 in each solution. This indicates the existence of conformer D in each solution.

**Figure 20:**
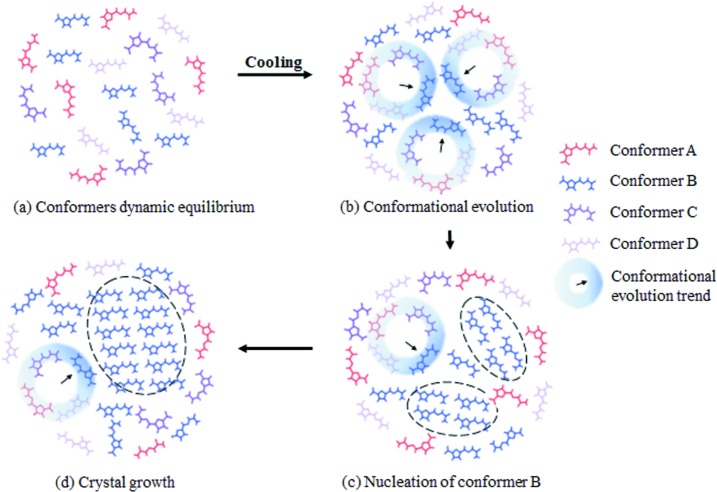
A schematic illustration of the crystallization process of 5-nitro­furazone form β.

All these data show that all 5-nitro­furazone solutions obtained by dissolving different polymorphs contain four conformers. However, the distributions of the four conformers among different solutions are different. Since this difference caused by different distributions of the four conformers is so subtle, the Raman spectra of solutions from the different forms (including different concentrations) are pretty similar. By referring to the above data and the concept of instinct supersaturation for single conformers in solutions containing multiple conformers, which was proposed by Derdour & Skliar (2012 ▸), the crystallization process of 5-nitro­furazone can be proposed, as schematically shown in Fig. 20 ▸. Different conformers keep in dynamic equilibrium in the solution [Fig. 20 ▸(*a*)], when temperature fluctuation destroys the dynamic equilibrium among conformers, one conformer could quickly transform into another one. During the crystallization process of 5-nitro­furazone, the conformer B reaches the instinct supersaturation first [Fig. 20 ▸(*b*)], since other conformers will transform into conformer B. Then, conformer B will nucleate from the solution and other conformers will continue to transform into conformer B to provide more conformer B molecules for further crystal nucleation and growth [Fig. 20 ▸(*c*)]. Consequently, crystals of conformer B will be continuously generated [Fig. 20 ▸(*d*)]. When the total supersaturation is exhausted by crystal growth, the crystallization process will stop and the conformers in the solution will turn back to the dynamic equilibrium.

### 3.5. Molecular conformational evolution during nucleation of crystals   

**Figure 21:**
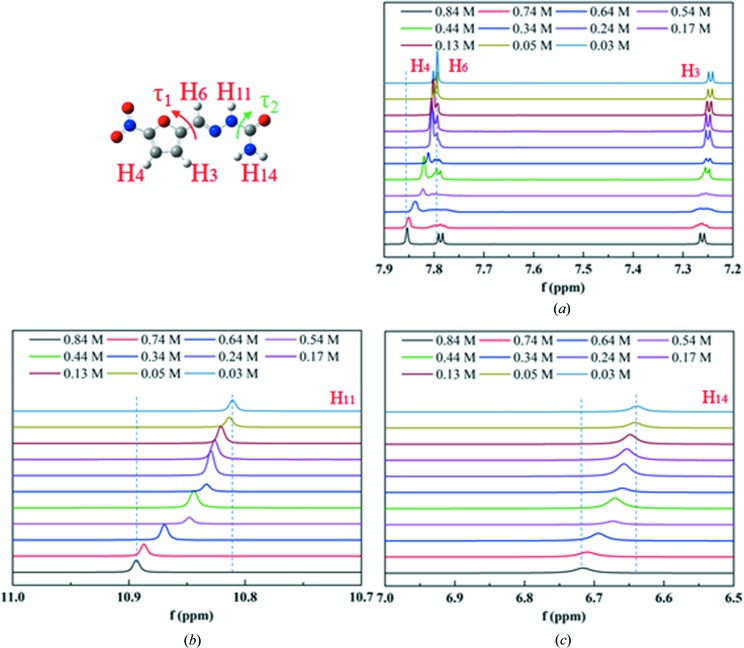
(*a*) ^1^HNMR spectra of H_3_, H_4_ and H_6_ at different concentrations. (*b*) ^1^HNMR spectra of H_11_ at different concentrations. (*c*) ^1^HNMR spectra of H_14_ at different concentrations.

It has been demonstrated by the above investigations that conformational evolution is involved in the nucleation of 5-nitro­furazone in solutions containing multiple conformers. ^1^HNMR spectra of DMSO solution of 5-nitro­furazone (taking form α as one example) with different concentrations were detected to deduce the conformational evolution path during nucleation of crystals [shown in Figs. 21 ▸(*a*)–21(*c*)].

The chemical shift of H_4_ [Fig. 20 ▸(*a*), 7.81–7.79 p.p.m.] moves to the high field with increasing concentration, which means that the intermolecular interaction decreases with increasing concentration. This illustrates that hydrogen-bond breakage related to H_4_ is faster than hydrogen-bond formation related to H_4_ with the increasing of concentration. Therefore, since hydrogen-bond breakage is mainly related to the desolvation process, the hydrogen-bond breakage of the desolvation process and corresponding conformational changes are faster than hydrogen-bond formation of solute molecular aggregation related to proton H_4_. In addition, between the concentrations of 0.74 to 0.34*M*, the peak of H_4_ becomes wider, which indicates that the conformation evolution related to H_4_ is slow in this concentration range. However, the peak of H_4_ becomes narrower when the concentration is lower than 0.34. This indicates that the speed of the desolvation process and its corresponding conformation changes is not linearly related with the concentration change. The chemical shift of H_3_ [Fig. 21 ▸(*a*)] does not show an obvious trend with the concentration increasing but shows a similar change of peak shape with the peak of H_4_. Therefore, the hydrogen-bond breakage of the desolvation process and its corresponding conformational evolution process and the hydrogen-bond formation of solute molecular aggregation related to proton H_3_ tend to be balanced. Generally, since the desolvation process is closely related to crystal nucleation, the changes of H_4_ and H_3_ are related to τ_1_ (the inter-transformation between conformers B and C and the inter-transformation between conformers D and A). Since the inter-transformation between conformers B and C and the inter-transformation between conformers D and A is easy, they will not directly decide nucleation conformation, although they can provide necessary conformation for the nucleation through conformational evolution.

The chemical shifts of H_6_ [Fig. 21 ▸(*a*), 7.80–7.85 p.p.m.], H_11_ [Fig. 21 ▸(*b*), 10.81–10.89 p.p.m.] and H_14_ [Fig. 21 ▸(*c*), 6.64–6.72 p.p.m.) move to the low field with increasing concentration. This indicates that the aggregation of solute molecules increases with increasing concentration. Therefore, H_6_, H_11_ and H_14_ are mainly related to the formation of interaction between solute molecules. The chemical shifts of these three protons are mainly affected by τ_2_, which is related to the transformation between A and C and the transformation between D and B. In addition, H_14_ is related to an intramolecular hydrogen bond (NH_2_⋯N) which should not change with the increasing concentration. However, it moves to the low field with the increasing of concentration, which shows that some intermolecular hydrogen bonds are formed in the solutions and the conformation could be further stabilized. Hence, the difficulty of conformer transformation will increase. Since the transformation between conformers A and C and the transformation between conformers D and B are difficult, these conformational transformation processes will affect nucleation conformation directly, and the different intermolecular interactions between different conformations will affect the polymorphic form of the crystal product. From conformation energy calculated by quantum chemical computation, it has been known that the conformational transformation direction for conformers A and C would be from conformer A to conformer C while the transformation direction for conformers B and D would be from conformer D to conformer B. They play the key roles in the selection of nucleation conformation. The inter-transformation between conformers B and C and the inter-transformation between conformers D and A, which are easy, will ensure the conformational balance to affect the nucleation indirectly. The formation of intermolecular interaction related to H_6_, H_11_ and H_14_ with the increasing of concentration might affect the polymorphic form of the crystal product, which makes the crystal product form β but not form γ structurally.

**Figure 22:**
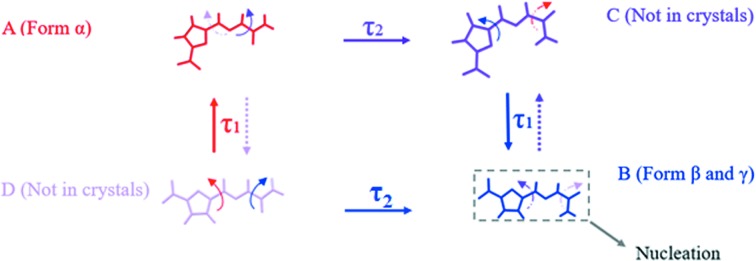
Schematic molecular conformational evolution during nucleation of 5-nitro­furazone form β.

To sum up the above analysis, the conformation evolution path during nucleation of 5-nitro­furazone form β in the solution is shown in Fig. 22 ▸. These four conformers all exist and keep dynamic equilibrium in the solution. The inter-transformation between conformers B and C and the transformation between conformers D and A always exist in the solution, and are easy and quick because of the similar conformational energy (<0.7 kcal mol^−1^) and lower energy barrier (<10 kcal mol^−1^). Generally, the conformer with higher energy tends to transform into a conformer with lower energy. Therefore, when temperature fluctuates, the transformation from conformer A to conformer C and the transformation from conformer D to conformer B will occur. Since the transformation from conformer C to conformer B is very quick and conformer C was not found in crystals, it can be reasonably deduced that conformer B would become supersaturated instantaneously and then conformer B would nucleate first. These results also confirm the crystallization process of 5-nitro­furazone form β proposed in Fig. 20 ▸ and the model proposed by Derdour’s investigations (Derdour *et al.*, 2011 ▸, 2012 ▸; Derdour & Skliar, 2012 ▸), in which the concept of ‘right conformer’ (the conformer which reaches the intrinsic supersaturation first and nucleates) in solutions containing multiple molecular conformations was put forward but not proved. The data presented in this work can explain why the specific conformer is the ‘right conformer’ and why the right conformer firstly reaches the intrinsic supersaturation from a structural point of view.

## 4. Conclusions   

Firstly, form α (conformational polymorphism) and form γ of 5-nitro­furazone were prepared through suspension experiments of form β. Cooling crystallization experiments were conducted and thermodynamic data were determined. Although Raman spectra of different solutions obtained by dissolving different forms of 5-nitro­furazone were pretty similar, 2D NOESY spectra, NMR data and molecular dynamics simulation results confirmed that all the solutions of 5-nitro­furazone obtained by dissolving different forms contain multiple conformers (four conformers). However, the distributions of different conformers were proven to be different among solutions obtained by dissolving different polymorphs. These four conformers keep in dynamic equilibrium in 5-nitro­furazone solutions. The conformational evolution and desolvation process was illustrated according to the ^1^HNMR spectra of different solutions with different concentrations. It was found that the conformers tended to transform into lower energy conformers when the temperature fluctuations destroyed dynamic equilibrium and hence the transformation from conformer A to conformer C and from conformer D to conformer B would occur. Since the transformation from conformer C to conformer B is very quick and conformer C was not found in crystals, it can be deduced that conformer B would become supersaturated first and hence nucleate first. Therefore, the results presented in this work give new insights into the molecular mechanism of nucleation of crystal from solution for conformational polymorphic systems. It can be used to regulate conformational polymorphism through inhibiting or promoting the corresponding conformation transformation.

## Supplementary Material

Supporting figures. DOI: 10.1107/S2052252520004959/yc5022sup1.pdf

## Figures and Tables

**Figure 13:**
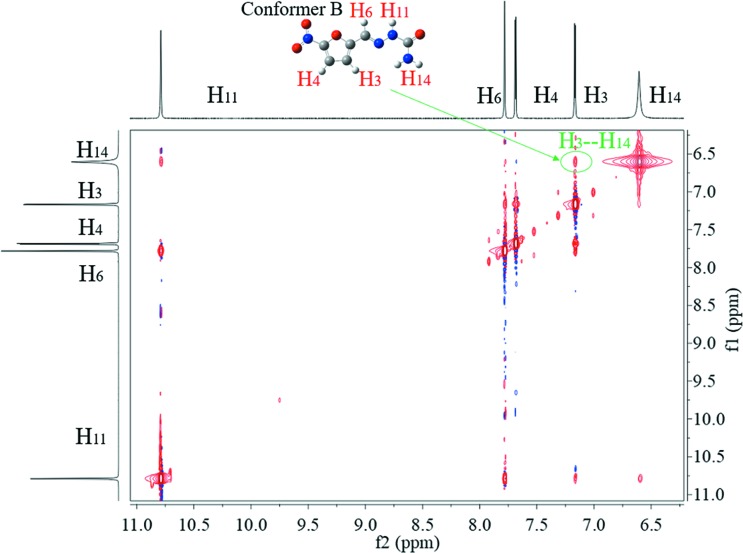
The 2D NOESY of form β saturated solution in DMSO at 20°C.

**Figure 14:**
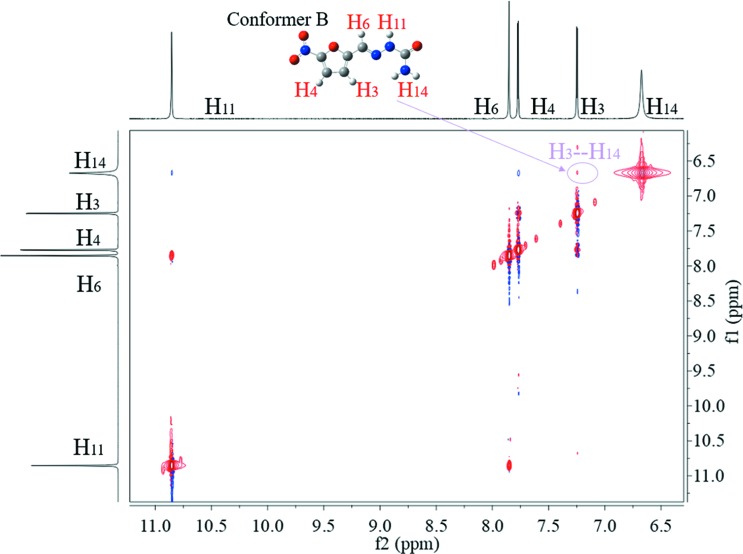
The 2D NOESY of form γ saturated solution in DMSO at 20°C.

**Table 1:** Interaction in three forms of 5-nitro­furazone

	Form α	Form β	Form γ
Chain structure	N—H⋯O=C	N—H⋯O=C	N—H⋯O=C
	C—H⋯O=C	NH_2_⋯O=C	C—H⋯O=C
	NH_2_⋯O=C	Intra NH_2_⋯N	Intra NH_2_⋯N
	Inter NH_2_⋯N		NO_2_⋯NH_2_
	NO_2_⋯NH_2_		NO_2_⋯H—C
	NH_2_⋯O		
Plane structure (links between chain structure)	NO_2_⋯H—C	NO_2_⋯H—C	NO_2_⋯H—C
Stacking of planar structures	NH_2_⋯O=C(dimer)	NO_2_⋯NH_2_	π⋯π
	π⋯π	π⋯π	

**Table 2:** The mass solubility of different forms in DMF, DMSO and DMAC at 20°C

	Form α (g/g)	Form β (g/g)	Form γ (g/g)
DMSO	0.1984 ± 0.0002	0.2379 ± 0.0003	0.1943 ± 0.0002
DMF	0.07553 ± 0.00005	0.08980 ± 0.00004	0.06844 ± 0.00003
DMAC	0.1023 ± 0.0003	0.1267 ± 0.0002	0.09843 ± 0.00002

**Table 3:** Characteristic distances of different conformers

	H_3_–H_14–1_(Å)	H_3_–O_3_(Å)	H_14–1_–O_1_(Å)	O_3_–O_1_(Å)	Characteristic distance
Conformer A	7.22	6.92	6.55	4.85	O_3_–O_1_
Conformer B	3.43	6.11	5.49	6.88	H_3_–H_14–1_
Conformer C	6.23	7.37	3.84	6.15	H_14–1_–O_1_
Conformer D	6.66	4.51	6.89	6.20	H_3_–O_3_

**Table 4:** The relative conformational energies in gas-phase and different solvent environments in SMD solvation models

	Energy (kJ mol^−1^)			
Solvent	Conformer A	Conformer B	Conformer C	Conformer D
Gas	9.21	0.00	0.07	6.26
DMF	2.70	0.66	0.00	2.97
DMSO	2.60	0.66	0.00	2.92
DMAC	2.70	0.66	0.00	2.97
