# Supplementary material for: Molecular conformational evolution mechanism during nucleation of crystals in solution
Source: IUCrJ. 2020 Apr 24;7(Pt 3):542–56. doi: 10.1107/S2052252520004959 (PMC7201291; doi:10.1107/S2052252520004959)
Supplement: Supplementary file 1 [file m-07-00542-sup1.pdf]

IUCrJ

Volume 7 (2020)

Supporting information for article:

Molecular conformational evolution mechanism during nucleation of crystals in solution

Xin Li, Na Wang, Jinyue Yang, Yunhai Huang, Xiongtao Ji, Xin Huang, Ting Wang, Honghai Wang and Hongxun Hao

S1. Single crystal structure of 5-nitrofurazone

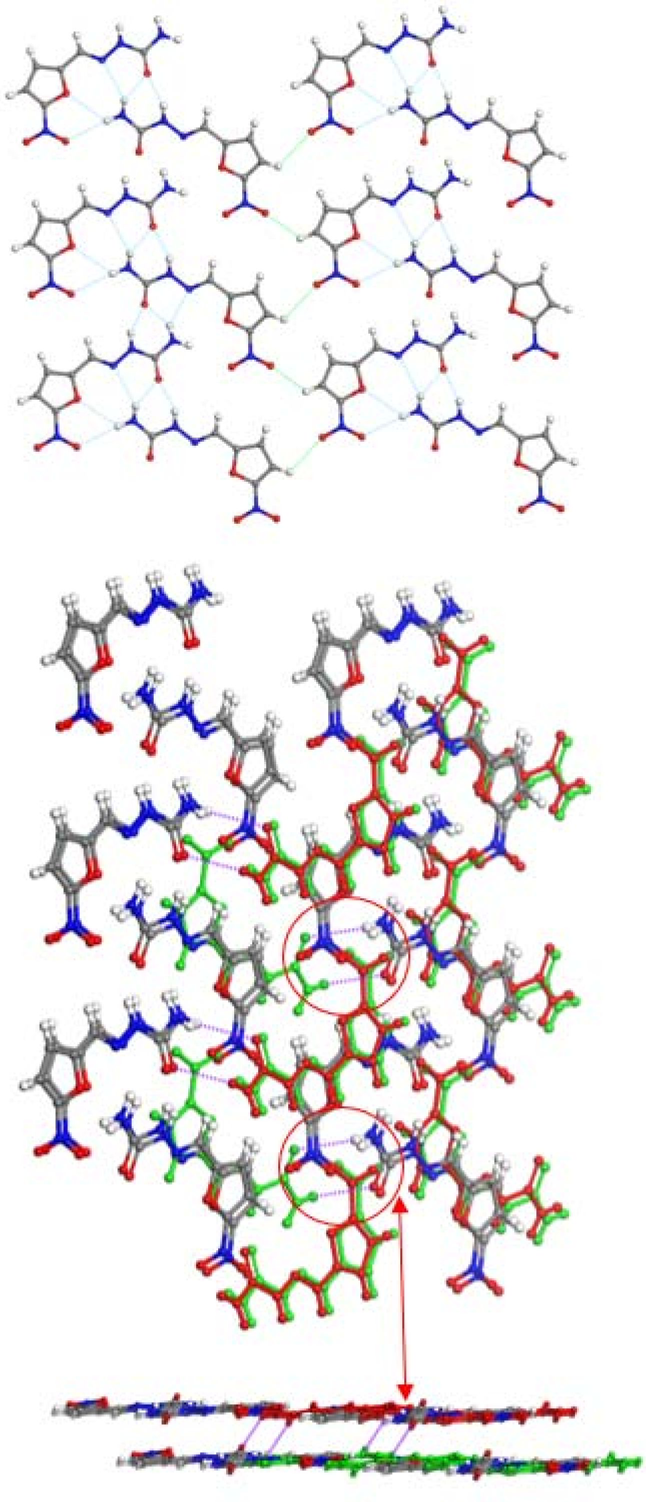

Figure S1 Single crystal structure of 5-nitrofurazone form α (CCDC references: 1292340).

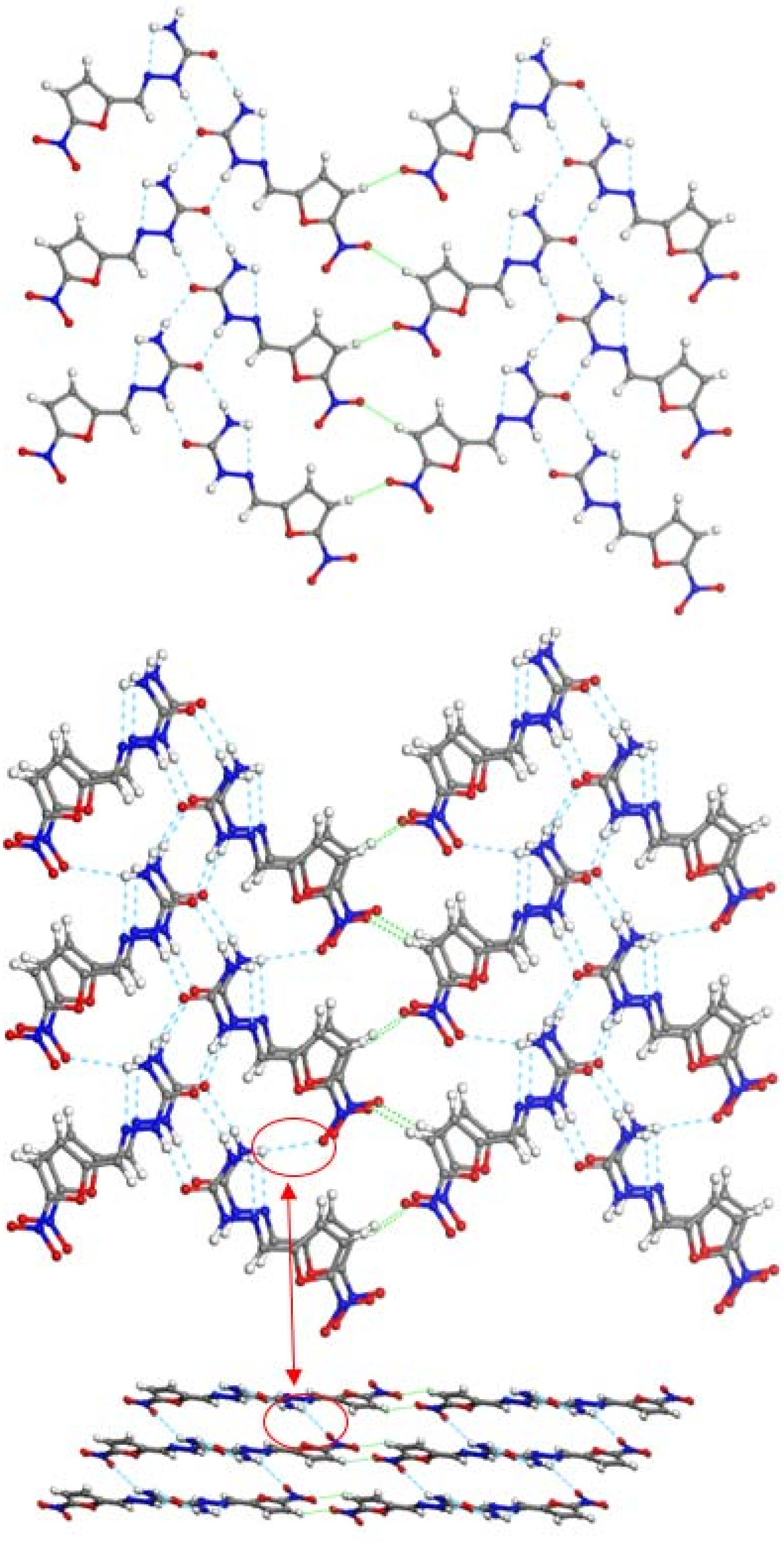

Figure S2 Single crystal structure of 5-nitrofurazone form β (CCDC references: 1444950).

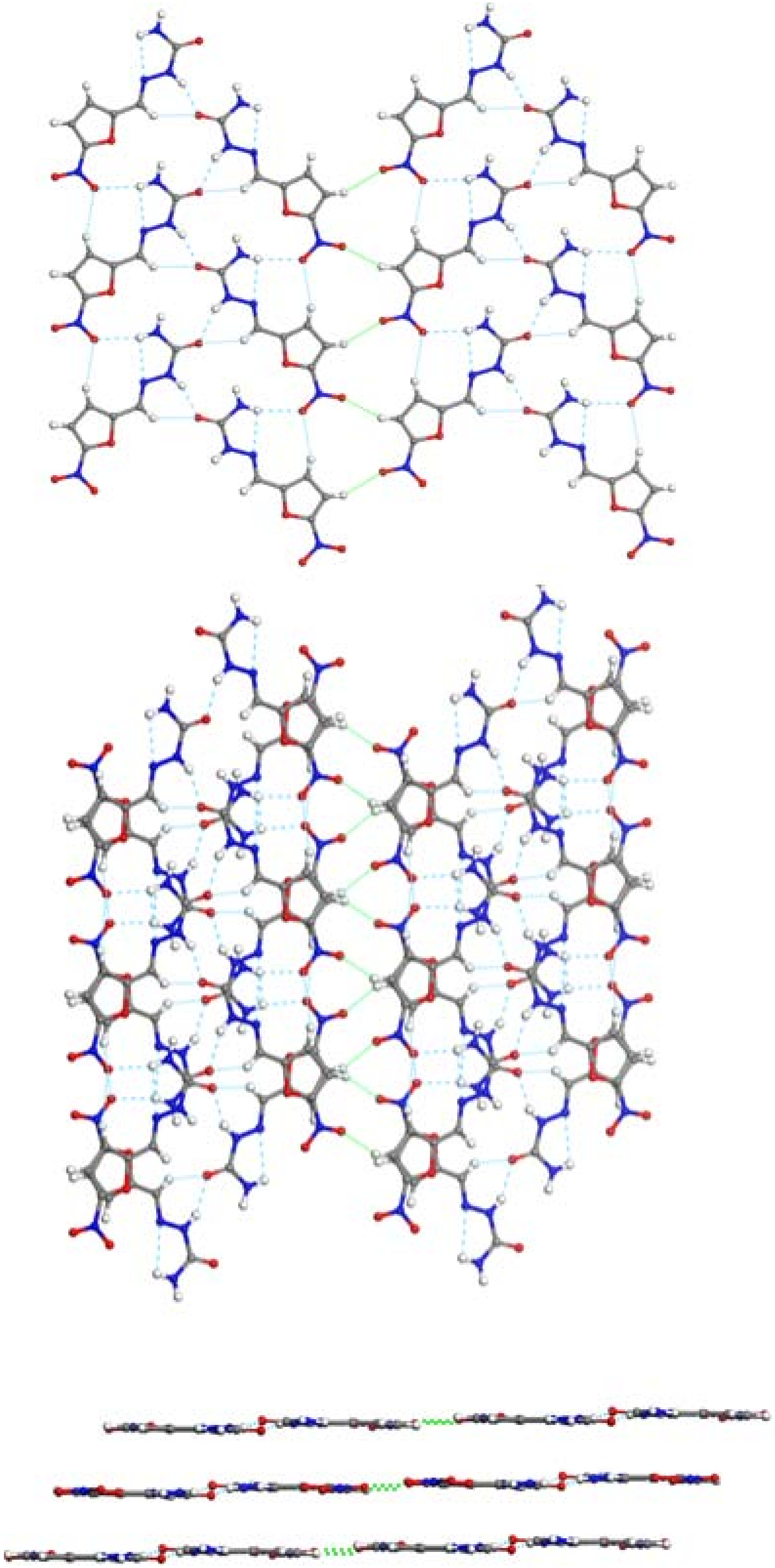

Figure S3 Single crystal structure of 5-nitrofurazone form γ (CCDC references: 1444951).
